# The Role of Predictive and Prognostic MRI-Based Biomarkers in the Era of Total Neoadjuvant Treatment in Rectal Cancer

**DOI:** 10.3390/cancers16173111

**Published:** 2024-09-09

**Authors:** Sebastian Curcean, Andra Curcean, Daniela Martin, Zsolt Fekete, Alexandru Irimie, Alina-Simona Muntean, Cosmin Caraiani

**Affiliations:** 1 Department of Radiation Oncology, Iuliu Hatieganu University of Medicine and Pharmacy, 8 Victor Babes Street, 400012 Cluj-Napoca, Romania; 2 Department of Radiation Oncology, ‘Prof. Dr. Ion Chiricuta’ Oncology Institute, 34-36 Republicii Street, 400015 Cluj-Napoca, Romania; 3 Department of Imaging, Affidea Center, 15c Ciresilor Street, 400487 Cluj-Napoca, Romania; 4 Department of Oncological Surgery and Gynecological Oncology, Iuliu Hatieganu University of Medicine and Pharmacy, 8 Victor Babes Street, 400012 Cluj-Napoca, Romania; 5 Department of Oncological Surgery, ‘Prof. Dr. Ion Chiricuta’ Oncology Institute, 34-36 Republicii Street, 400015 Cluj-Napoca, Romania; 6 Department of Medical Imaging and Nuclear Medicine, Iuliu Hațieganu University of Medicine and Pharmacy, 400012 Cluj-Napoca, Romania

**Keywords:** magnetic resonance imaging, rectal cancer, MRI-based biomarkers, watch-and-wait, total neoadjuvant treatment

## Abstract

**Simple Summary:**

Magnetic resonance imaging (MRI) plays a crucial role in rectal cancer management, offering valuable information for staging, treatment response, and patient prognosis. MRI biomarkers, such as circumferential resection margin (CRM), extramural venous invasion (EMVI), tumour deposits, and MRI tumour regression grade (mrTRG) alongside functional imaging techniques such as diffusion-weighted imaging (DWI) and dynamic contrast enhancement (DCE) are essential in clinical decision-making. Additionally, emerging technologies like radiomics and artificial intelligence (AI) are showing promise in improving the precision of rectal cancer care. As the focus increasingly shifts toward non-invasive management, such as ‘watch-and-wait’ approach, this review discusses the role of predictive and prognostic MRI biomarkers in rectal cancer and how they integrate into everyday clinical practice.

**Abstract:**

The role of magnetic resonance imaging (MRI) in rectal cancer management has significantly increased over the last decade, in line with more personalized treatment approaches. Total neoadjuvant treatment (TNT) plays a pivotal role in the shift from traditional surgical approach to non-surgical approaches such as ‘watch-and-wait’. MRI plays a central role in this evolving landscape, providing essential morphological and functional data that support clinical decision-making. Key MRI-based biomarkers, including circumferential resection margin (CRM), extramural venous invasion (EMVI), tumour deposits, diffusion-weighted imaging (DWI), and MRI tumour regression grade (mrTRG), have proven valuable for staging, response assessment, and patient prognosis. Functional imaging techniques, such as dynamic contrast-enhanced MRI (DCE-MRI), alongside emerging biomarkers derived from radiomics and artificial intelligence (AI) have the potential to transform rectal cancer management offering data that enhance T and N staging, histopathological characterization, prediction of treatment response, recurrence detection, and identification of genomic features. This review outlines validated morphological and functional MRI-derived biomarkers with both prognostic and predictive significance, while also exploring the potential of radiomics and artificial intelligence in rectal cancer management. Furthermore, we discuss the role of rectal MRI in the ‘watch-and-wait’ approach, highlighting important practical aspects in selecting patients for non-surgical management.

## 1. Introduction

Magnetic resonance imaging (MRI) plays a central role in the management of rectal cancer, offering morphological and functional information that extends beyond traditional TNM staging. Rectal MRI has become a key pillar in the clinical diagnostic framework, particularly for staging, response evaluation, and follow-up of rectal cancer patients [1]. The primary treatment strategies for locally advanced rectal cancer include neoadjuvant chemoradiotherapy followed or not by total mesorectal excision (TME) surgery, with the indication mainly guided by TNM stage. Morphological MRI features such as T staging, circumferential resection margin (CRM), extramural venous invasion (EMVI), tumour deposits (TD), and mucinous differentiation are essential for clinical decision-making and should be documented in rectal cancer staging MRI reports [2]. The expanding role of imaging biomarkers includes detection of high-risk tumour features, prediction of response to neoadjuvant treatment, and patient prognosis, which may assist multidisciplinary teams in personalising treatment options.

Over the last decade, functional imaging has gained significant momentum, with new imaging biomarkers providing insights into the tumour microenvironment. MRI-based biomarkers derived from diffusion-weighted imaging (DWI) or dynamic contrast-enhanced MRI (DCE-MRI) correlate with pathological features such as cellular density, amount of necrosis, and tumour vascularity [3,4]. Radiomics and texture analysis have shown potential across various areas of rectal cancer management, including staging, prediction of response to neoadjuvant therapy (NAT), prognosis, tumour aggressiveness, and genetic characteristics. Their precision is enhanced by artificial intelligence (AI), which uses machine learning (ML) techniques such as convoluted neural networks (CNNs) or deep learning (DL) [5]. Radiogenomics links imaging with genetic profiles, predicting genotypic features such as KRAS status and microsatellite instability, essential in guiding targeted therapy.

Total neoadjuvant treatment (TNT) has dramatically increased the rate of complete response, challenging the traditional surgical approach in the pursuit of organ preservation. The rate of pathological complete response has risen from the previously reported 10–30% to as high as 60% [6]. Deferring surgery reduces morbidity and increases quality of life without impacting overall survival. MRI plays a pivotal role in the ‘watch-and-wait’ strategy, distinguishing between complete (or near-complete) responses that may be suitable for active surveillance from residual tumours that require surgery. The combination of clinical and imaging assessments is particularly important, as the gold standard of pathological analysis is not available in this setting [7]. The quality of MRI reports is enhanced by standardized proformas, which provide consistent information relevant to clinicians in rectal cancer staging [8]. This review aims to describe validated morphological and functional MRI-derived biomarkers with prognostic and predictive value while also exploring the potential of radiomics and artificial intelligence in rectal cancer care. Additionally, we address the role of rectal MRI in the ‘watch-and-wait’ approach as the focus shifts from surgical to non-surgical management.

## 2. Conventional MRI

### 2.1. Tumour Extent beyond Muscularis Propria

Tumour extension within the mesorectum (in millimetres) defines T3 subcategories as they influence survival rates. Four subcategories have been proposed: T3a—tumour extension beyond the muscularis propria (MP) not exceeding 1 mm; T3b—1–5 mm beyond the muscularis propria; T3c—5–15 mm beyond the muscularis propria; T3d—more than 15 mm beyond the muscularis propria [9]. Empirically, the T3 stage was treated using neoadjuvant chemoradiation regardless of the subcategory. However, tumours spreading ≤5 mm into the mesorectum have local recurrence and survival rates similar to those limited to the muscularis propria (T2) [9]. Therefore, current European guidelines recommend total mesorectal excision (TME) surgery alone for early rectal cancer patients (≤T3b stage, negative CRM), with NAT followed by TME proposed only for locally advanced cancer patients (≥T3b, positive CRM, or presence of extramural venous invasion) [9]. High-resolution MRI accurately measures the depth of extramural tumour spread and corresponds to histopathological assessments within 0.5 mm, as demonstrated by the MERCURY study group [10]. Subsequent data reported a pooled MRI sensitivity and specificity of 87% and 75% for T staging [11], consistent with a recent study showing 85% sensitivity and 72% specificity [12]. For muscularis propria invasion, the sensitivity and specificity were 97% and 58%, respectively [12]. A clinical trial found MRI staging matched histopathological staging in 63.5% of cases, with a 22.9% rate of overstaging [13]. In the post-neoadjuvant therapy setting, the accuracy of high-resolution T2-weighted imaging (T2WI) for evaluating T staging of rectal cancer was 49.57%, likely due to limitations in assessment caused by inflammatory response, fibrosis, and mucinous degeneration [14]. In addition, the technique can assess CRM and EMVI status, differentiating between low-risk and high-risk disease, making high-resolution MRI the method of choice for primary staging and restaging after neoadjuvant chemoradiotherapy.

### 2.2. Circumferential Resection Margin (CRM)

**Figure 1:**
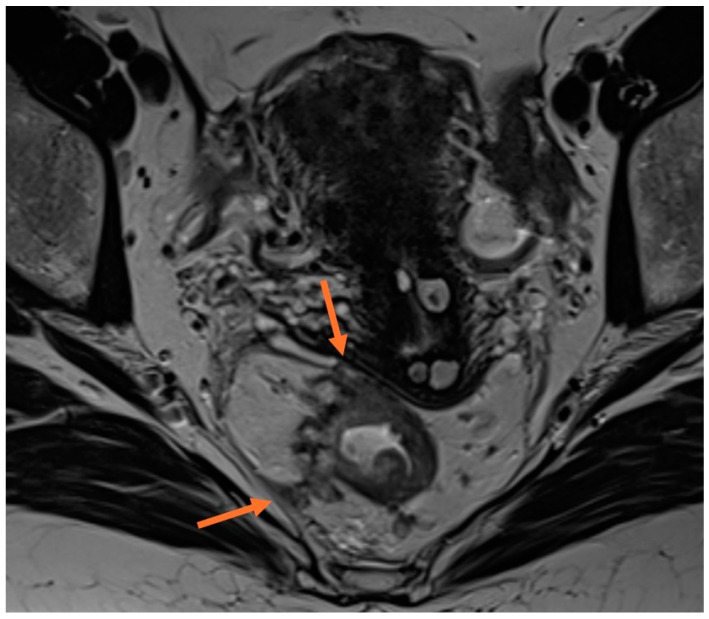
Axial T2-weighted MRI illustrating rectal tumour infiltrating all layers of the rectal wall, with evidence of mesorectal fascia involvement (orange arrow).

MRI CRM status in rectal cancer is the strongest imaging biomarker to predict local recurrence and a reliable prognostic factor for disease-free survival and overall survival. The outcome of standard total mesorectal excision (TME) surgery depends on the tumour’s relationship to the mesorectal fascia. A positive CRM is defined as a distance of 1 mm or less between the tumour or irregular lymph nodes and the mesorectal fascia, as agreed upon in guidelines by the European Society of Gastrointestinal and Abdominal Radiology (ESGAR) [2] and the North American Society of Abdominal Radiology (SAR) [15]. Figure 1 shows a positive CRM on axial T2 images. The MERCURY study demonstrated that MRI is an accurate tool for assessing CRM status, with a specificity of 91%, a negative predictive value of 94%, and an accuracy of 87% in predicting clear margins, using histopathology as the reference [10]. The local recurrence rate in CRM-positive patients was 20%, compared to 7% in CRM-negative patients. A follow-up study showed that the 5-year overall survival (OS) was 62.2% versus 42.2% in MRI-negative CRM versus MRI-positive CRM patients, respectively, having a greater impact than T and N staging [16]. The results of a recent clinical trial assessing the diagnostic accuracy of MRI for predicting CRM involvement indicated an accuracy of 86.5%, with a negative predictive value of 98.1%, using a cut-off of >1 mm for predicting a negative CRM [13]. MRI accuracy in evaluating CRM status was also validated in prospective multicentre studies [17].

At initial staging, CRM status, alongside TNM staging, contributes to better patient stratification and a more tailored treatment approach. Patients with a negative CRM may benefit from primary surgery alone, avoiding the morbidity associated with neoadjuvant chemoradiotherapy, without impacting the rate of local and systemic recurrence in early-rectal cancer [9]. The prospective Quicksilver study selected patients with MRI criteria for ‘good prognosis’ to undergo primary surgery and found low rates of positive CRM on pathological specimens [18]. These criteria included a tumour distance from the mesorectal fascia greater than 1 mm, stage T3b or less, the absence of discontinuous tumour nodules or suspicious lymph nodes and absent or equivocal extramural venous invasion. The study concluded that MRI is a safe and reliable tool for selecting patients eligible for primary surgery, avoiding the need for neoadjuvant treatment (NAT) [18]. The RAPIDO trial successfully used MRI-based criteria (including CRM status) to select high-risk patients for short-course radiotherapy followed by chemotherapy and delayed surgery [19]. CRM status at diagnosis also has predictive value for tumour response to NAT, with positive CRM being linked to a poor response to NAT [20]. 

Following neoadjuvant chemoradiation, some studies found that MRI-detected positive CRM correlates well with histopathologic CRM status [21]. Post-treatment fibrotic changes may alter detection of residual tumour, with pathologic discrepancies; therefore MRI should not be used as a single tool in decision-making after NAT [22]. Nevertheless, MRI still remains an accurate tool to assess CRM involvement after neoadjuvant therapy, guiding a more radical surgical approach in patients with predicted positive margins [23].

ESGAR guidelines recommend including the location of the tumour with respect to the anterior peritoneal reflection in the structured MRI report. For tumours located below the anterior peritoneal reflection, CRM status should be included [2]. In low rectal cancers involving the anal canal, there is minimal mesorectal fat separating the tumour from the external sphincter; therefore, the relationship of the tumour to the intersphincteric plane is of utmost importance. Both mesorectal fascia and intersphincteric plane must be tumour-free for a curative treatment. Even tumours involving the full thickness of the muscularis propria are at high risk of positive CRM; therefore, a beyond TME approach such as extralevator abdominoperineal excision (APE) should be taken into consideration to minimise the risk of positive resection. The MERCURY II study prospectively validated pre-operative MRI assessment in low rectal cancer patients, both at initial staging and after neoadjuvant treatment [24].

### 2.3. Extramural Venous Invasion (EMVI)

Extramural venous invasion (EMVI) is a strong predictor of disease recurrence, particularly systemic failure [25,26,27,28], as well as for synchronous and metachronous metastases [28]. Furthermore, a study showed that about 90% of patients with hepatic metastases are EMVI positive [29]. Positive EMVI correlates with reduced disease-free survival (DFS), with EMVI-positive stage II disease showing similar outcomes to those of EMVI-negative stage III disease [30]. Additionally, EMVI status has demonstrated a higher prognostic value compared to current T and N staging [31] and has shown good correlation with histopathological findings [32]. Recent studies suggest that MRI-detected EMVI correlates with poorly differentiated tumours and may serve as an imaging biomarker for nodal metastases [33].

In light of robust, standardised, and validated methods used to assess local tumours using MRI, ESGAR currently recommends documenting EMVI status during both initial staging and restaging after NAT, as supported by NICE [34] and NCCN guidelines [35]. This approach allows for preoperative patient stratification into low-risk or high-risk categories and facilitates a more tailored treatment strategy for a significant proportion of the rectal cancer patient population, with the mean EMVI prevalence of 26% reported in a systematic review [36]. MRI-detected EMVI (mrEMVI) status prior to chemoradiation can help identify T3 rectal cancer patients who may benefit from neoadjuvant chemoradiation [37]. MRI may also contribute to more accurate gross tumour volume delineation by including EMVI areas in radiotherapy planning, thereby enhancing the delivery of high-dose radiotherapy to macroscopic disease [38].

**Figure 2:**
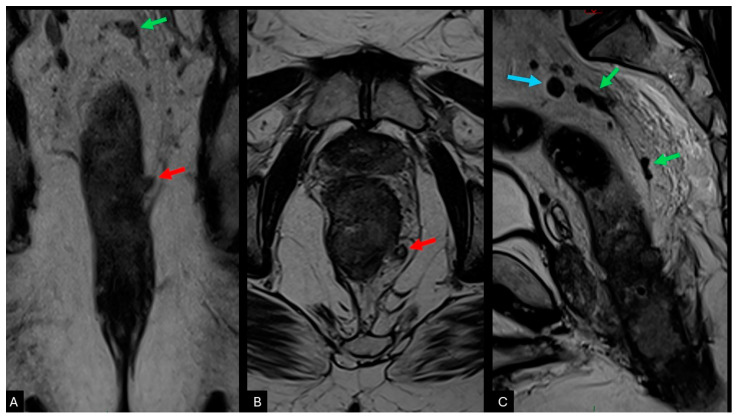
T2-weighted MRI in three planes illustrating a low rectal tumour infiltrating all layers of the rectal wall, with evidence of extramural venous invasion (EMVI, red arrow), tumour deposits (green arrow), and malignant lymph nodes (blue arrow). (**A**) Coronal T2WI showing extramural venous invasion (red arrow) with increased vessel calibre, maintaining contact with the primary tumour; tumour deposits (green arrow) located superiorly tapering into a vessel but discontinuous from the primary tumour. (**B**) Axial T2WI showing increased vessel calibre and loss of the normal flow void in the mesorectal vein adjacent to the tumour, suggestive of EMVI (red arrow). (**C**) Comparison between tumour deposits (green arrow), which are inseparable from the course of veins, and lymph nodes (blue arrow), which are isolated by surrounding fat.

Emerging evidence of the importance of EMVI has led to the standardisation of definitions for mrEMVI in the preoperative evaluation of rectal cancer as ‘serpiginous tumour signal extension within a vascular structure, leading to either contiguous or discontinuous tumour signal expansion of a vein [39,40]. On T2-weighted images (T2WI), EMVI is visible as an intermediate signal within the mesorectal vessels, with a loss of normal flow void on T2WI; additional features include irregular contours and increased calibre of the mesorectal vessels (Figure 2). Traditionally, T2WI was the workhorse sequence for detecting EMVI; however, some studies suggest that DWI may increase EMVI detection after NAT [41]. Further studies explored the clinical implications of positive EMVI after NAT and potential correlation with patients outcomes [42]. Recent findings suggest that disease detected using DWI is also correlated with poor prognosis [43]. Beyond its high prognostic value, EMVI status may also predict response to NAT, with positive EMVI being associated with a lack of tumour response [20]. MRI shows good performance in diagnosing EMVI, with a sensitivity of 77.0% and specificity of 85.8%, and substantial agreement between readers of varying experience levels [44]. Additionally, a meta-analysis evaluating the diagnostic performance of MRI for EMVI detection showed a pooled sensitivity of 0.61 (95% CI: 0.49–0.71) and a pooled specificity of 0.87 (95% CI: 0.79–0.92) [45].

After neoadjuvant chemoradiation, fibrotic changes may impede the detection of EMVI on pathological specimens following surgery, with better detection reported on MRI [40,46]. The Royal College of Pathologists in the UK suggests that MRI should be utilised to improve detection rates in pathological analysis, as pathology has a higher rate of underdiagnosis due to sampling [1]. The sensitivity and specificity of MRI detection of EMVI after neoadjuvant therapy (yMR-EMVI) were 76.19% and 79.75%, respectively [47]. Positive EMVI after NAT is associated with poor outcomes and higher rates of local and systemic failure; therefore, its detection is of utmost importance, as it may influence subsequent treatment and follow-up strategies [48,49]. Recent data from a study of 1184 patients show that EMVI status is a predictive biomarker of poor disease-free survival (DFS) and systemic recurrence (SR) following NAT in locally advanced rectal cancer [50]. Another study suggested that adjuvant chemotherapy has a positive impact on DFS in EMVI-positive patients after NAT [51]. Additionally, other results showed similar outcomes in patients with EMVI regression to those with negative EMVI at baseline [52]. Therefore, EMVI detection following NAT may significantly impact treatment strategies, potentially prompting a more aggressive approach for EMVI-positive cases.

### 2.4. Tumour Deposits (N1c)

Tumour deposits (TD) are defined as irregular nodules located within the mesorectal fat, discontinuous from the primary tumour. The prevalence of TD ranges from 10.2% to 44.2%, with a median of 21.3% in a meta-analysis [53]. TD were pathologically described in patients with negative lymph nodes, suggesting that non-lymphatic deposits were caused by haematogenous spread. Additionally, the presence of TD correlates with positive EMVI, supporting the vascular spread mechanism [53]. Due to varying definitions used in TNM classifications, there remains a significant overlap between TD and malignant lymph nodes. These classifications have evolved from using size as the main criterion (nodules over 3 mm were considered lymph nodes, while those smaller than 3 mm were noted as discontinuous tumour extension—T3) to morphological criteria (lack of smooth contour and nodal structure), leading to the definition used in the 7th edition, where the N1c category was proposed without changes to the T category. The most recent 8th edition defines tumour satellites as ‘discrete macroscopic or microscopic nodules of cancer in the pericolorectal adipose tissue’s lymph drainage area of a primary carcinoma that are discontinuous from the primary and without histological evidence of residual lymph node or identifiable vascular or neural structure’ [54]. Efforts to differentiate tumour deposits (TDs) from lymph node metastases (LNMs) on MRI have been recently initiated. TDs are considered irregular nodules within the mesorectum adjacent to vascular channels, but not within a vessel as in EMVI [39]. This is illustrated in Figure 2. Compared to malignant lymph nodes, TDs are inseparable from the vein and tend to taper into the vessel (comet-tail appearance), rather than describing an acute angle with the vein [31].

There is increasing evidence that TDs are an imaging biomarker linked to poor prognosis, with an impact on overall survival [55,56,57,58,59]. Similarly, TDs are also correlated with poor outcomes in patients previously treated with NAT [60]. As previously mentioned, recent studies suggest that current T and N staging is less accurate in predicting prognosis compared to MRI-detected tumour deposits and EMVI [31]. However, it remains unclear whether the presence of TD should be recorded as a high-risk feature [61]. Further studies are needed to assess the clinical implications of N1c staging. 

Whilst MRI has proven to be an accurate tool for EMVI detection, validated data confirming the accurate detection of tumour deposits are scarce. Most studies assess the presence of tumour deposits in conjunction with EMVI [58,59,60], as both involve tumours spreading through adjacent veins. This may be due to a lack of definition concordance and inconsistent pathology reporting. To our knowledge, there is a single multicentre prospective clinical trial aiming to address MRI detection of tumour deposits against pathological specimens, the gold standard [62]. Similar to the MERCURY trial, the COMET trial aims to demonstrate the negative impact of TD on patient outcomes, comparable to that of EMVI. It is hypothesised that MRI detection of TDs will improve pathological detection and demonstrate that they are part of the same process as EMVI, with the results being eagerly awaited.

It has been suggested that DWI in combination with DCE-MRI may help differentiating between metastatic lymph nodes and TD with 71.19% accuracy, 86.70% sensitivity, 55.20% specificity, 66.67% positive predictive value, and 80% negative predictive value [63]. Kim T.H. et al. showed evidence that the same is true in the NAT setting [43]. Radiomic models have also been proposed to predict tumour deposits and lymph node metastases [64,65,66,67]. However, further research is needed to validate these findings.

### 2.5. Lymph Nodes

**Figure 3:**
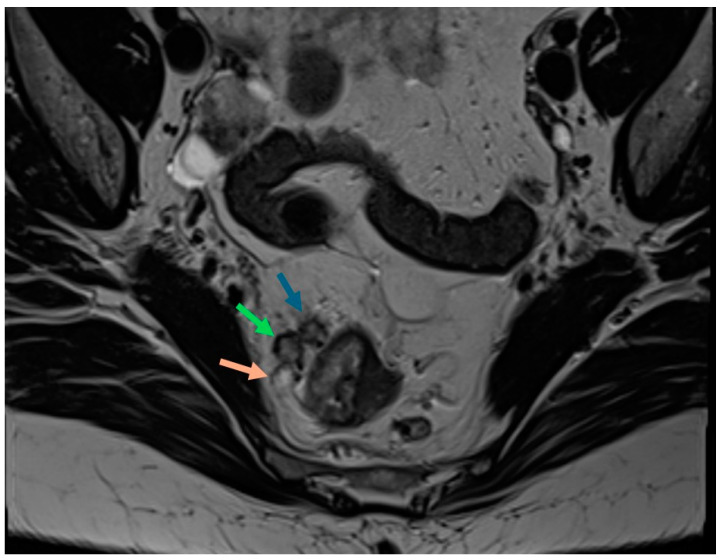
Axial T2-weighted MRI demonstrates malignant morphological features of mesorectal lymph nodes, including a round shape and heterogeneous internal signal (green arrow), irregular margins (blue arrow), and mucinous component (orange arrow).

The ESGAR guidelines for nodal staging include traditional size and morphological criteria as the most reliable imaging features for N staging. These criteria include a short axis measuring ≥9 mm, 5–8 mm with ≥2 morphologically suspicious features, or <5 mm with 3 morphologically suspicious features. Morphological characteristics include a round shape, irregular margins, and heterogeneous internal signal. All mucinous lymph nodes are considered malignant [2]. This is illustrated in Figure 3. These features are best visualised using a lower field of view (16 cm × 16 cm). These morphological MRI criteria remain limited in identifying malignant lymph nodes both at staging and restaging after NAT [68]. In a meta-analysis of thirty-seven studies, the pooled sensitivity, specificity, and diagnostic odds ratio of preoperative MRI for lymph node staging were 0.73 (95% CI: 0.68–0.77), 0.74 (95% CI: 0.68–0.80), and 7.85 (95% CI: 5.78–10.66), respectively, with a diagnostic accuracy ranging from 0.75 to 0.81 [69]. A subsequent study found that combining morphologic criteria with radiomic features improved diagnostic accuracy, achieving a sensitivity of 72.2%, specificity of 91.1%, and accuracy of 82.8%, compared to 75%, 80%, and 77.8% for morphologic criteria alone [70]. In the post-NAT setting, for patients with pathological complete response, results showed a sensitivity of 37%, specificity of 84%, positive predictive value of 70%, and negative predictive value of 57% for identifying the presence of residual regional lymph node metastases [71].

Nodal spread is considered an important factor in local tumour recurrence and represents a primary indication for neoadjuvant therapy (NAT). However, some studies argue that lymph node involvement has limited impact in the era of total mesorectal excision, with the source of potential recurrence being removed ‘en bloc’ alongside the mesorectal fascia and fat [72,73]. Tumours with low-risk MRI features (less than T3c staging, negative CRM, and negative EMVI) show a low risk of tumour recurrence regardless of nodal involvement [74]. An additional study emphasised that positive lymph nodes located adjacent to the mesorectal fascia are rarely the cause of a positive resection margin [75]. Overstaging lymph nodes may increase morbidity secondary to chemoradiation with minimal impact on reducing local recurrence.

Positive lymph nodes may be mistaken for TD if the tumour involves the nodal capsule. In contrast to N+ stage, T1c stage represents a biomarker for poor prognosis; therefore, differentiation between the two is key. Lymph nodes are usually isolated within the mesorectal fat, whilst tumour deposits follow the course of a venous channel (Figure 2). A recent multidisciplinary expert consensus agreed that any discontinuous nodules from the main tumour should be regarded as N+ category until results of COMET trial would help clarifying this issue [76]. 

Tumour extension beyond the nodal capsule, known as extranodal tumour extension, has been shown in recent studies to be reliably detected using MRI during staging and is associated with poor prognosis [77,78]. A T2-based radiomics model has also been developed to predict extranodal extension [79].

Regional lymph nodes are considered to include mesorectal nodes, distal sigmoid mesocolon, and lateral pelvic lymph nodes (obturator, internal, and external iliac). Reporting specific lymph node stations is relevant for both radiotherapy and surgical planning. Lymph nodes coursing along the superior rectal vessels should be included in the radiotherapy field [80]. Obturator and internal iliac nodes are associated with a high risk of local failure, which can be prevented by either surgical lymph node resection [81] or targeted radiotherapy [82,83]. Lymph nodes outside of these areas are considered metastatic. Of note, inguinal nodes are regarded as regional lymph nodes in low rectal cancers. Previous studies have shown that positive external iliac nodes are associated with increased rates of systemic failure rather than local relapse [84]. Lymph node maps improve consistency in MRI reporting, and thus an adapted scheme was proposed by recent multidisciplinary expert consensus [76]. Further evidence is needed to define criteria for positive lymph nodes after chemoradiation.

### 2.6. Mucinous Tumours

**Figure 4:**
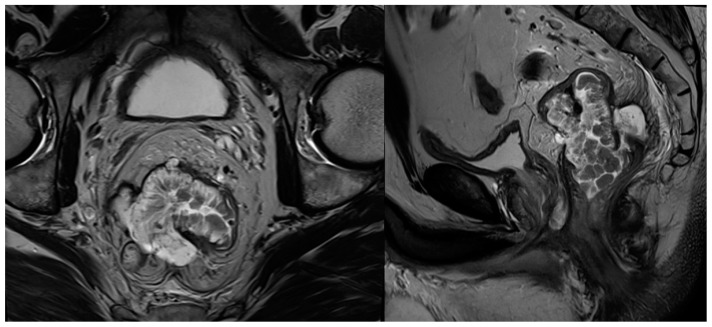
T2-weighted MRI in axial and sagittal planes of a rectal tumour demonstrating extensive high-T2 signal mucinous component.

Mucinous adenocarcinomas are associated with poor disease-free and overall survival and seem to be more resistant to NAT [85,86]. Therefore, highlighting tumours containing mucin may guide treatment approach before pathologic specimen is available. Mucin components can be reliably identified on MRI as areas of increased T2 signal within the tumour with a sensitivity and specificity of up to 100% and 98%, respectively [87]. Figure 4 depicts a mucinous adenocarcinoma of the rectum. Mucinous degeneration occurs after neoadjuvant therapy and may be a sign of treatment response known as ‘colloid degeneration’ [88], but not of complete response [86]. Recent studies suggested that diffusion-weighted MRI imaging may be useful in detection of mucus pool after radiochemotherapy and may represent an imaging biomarker for mucinous adenocarcinoma response to treatment [88]. Mucinous degeneration assessment using MRI remains challenging, since acellular mucin pools cannot be differentiated from cellular mucinous tissue. 

### 2.7. MRI Tumour Regression Grade (mrTRG)

mrTRG is an adapted imaging scoring system which is based on the histopathological grading system in post-neoadjuvant treatment (NAT) settings. It grades the amount of fibrosis and residual tumour detected on T2WI in a 1-5 scale. mrTRG 1 represents lack of intermediate tumour signal replaced by hypointense fibrotic signal, corresponding to complete response. Histopathological tumour regression grade is a well-established biomarker for patient outcomes [89]. mrTRG has also established its role as an important prognostic factor for overall survival, surpassing ymrT restaging, as demonstrated by the MERCURY study [90]. The emerging organ-preservation strategies require identification of complete responders to select eligible patients for active surveillance deferring surgical intervention. The main limitation of tumour reassessment after NAT is distinguishing residual tumour from fibrotic changes or desmoplastic reaction. Research suggests that complete response on MRI does not correspond to pathological complete response; however, it may indicate inactive tumours with decreased likelihood of regrowth [91]. MRI-based tumour regression grades frequently fail to accurately predict pathologic tumour regression grades, with accuracy ranging from 28% to 34%, showing equal rates of under- and overestimation. Sensitivity and specificity of mrTRG 1-2 (complete/good radiological regression) for the prediction of pathological complete response was 74.4% (95% CI: 58.8–86.5), and 62.8% (95% CI: 54.5–70.6), respectively [92]. A recent prospective study reported a sensitivity, specificity, and accuracy of mrTRG 1-3 to predict good pathological response of 100%, 46.3%, and 62.7%, respectively [93]. The outcomes of a prospective study, the TRIGGER trial, which hypothesise that mrTRG 1-2 scores correlates with lack of tumour recurrence over a long-time interval and suitability for ‘watch-and-wait’ approach, are eagerly awaited [94]. A recent study performed on 83 patients at Karolinska Institute concluded that mrTRG1 is correlated with a sustained clinical complete response and lack of tumour recurrence in the first two years [95]. Even though mrTRG score does not reflect pathological changes, it still plays an important role in identifying complete/near complete responders with low likelihood of local recurrence amenable to the ‘watch-and-wait’ approach.

## 3. Functional MRI

### 3.1. Diffusion Weighted Imaging (DWI)

DWI measures cellular density by evaluating water molecule diffusion in tissues. The apparent diffusion coefficient (ADC), derived from DWI, inversely correlates with tumour cellularity: viable tumour cells reduce water diffusion, causing low ADC values, while necrosis increases water diffusion, resulting in higher ADC values [4]. 

Over the last ten years, research on the use of diffusion-weighted imaging (DWI) in rectal cancer has increased exponentially. The use of DWI is multifaceted, playing multiple roles across different stages of rectal cancer management. The routine use of DWI is included in the ESTRO guidelines for both primary staging and reassessment after neoadjuvant therapy (NAT) [2]. The role of DWI in tumour detection and staging is limited, with no additional value in T-staging of rectal cancer; however, it may highlight areas of interest in small tumours. No additional value was found for EMVI detection at staging [96], although quantitative data suggest that low ADC values are correlated with EMVI-positive tumours [97]. During restaging, DWI may be useful in detecting viable extramural venous invasion (EMVI) and tumour deposits (TDs). Using a five-point Likert scale, DWI detection showed high specificity (96%) but only moderate sensitivity (55%) for identifying viable EMVI and TDs after NAT [43] While DWI may improve lymph node detection at staging, it cannot distinguish between benign and malignant lymph nodes [98,99]. However, a recent meta-analysis reported a sensitivity of 81% and specificity of 74% in distinguishing between metastatic and non-metastatic lymph nodes, with a positive predictive value of 63%, negative predictive value of 85%, and an accuracy of 82% [100]. 

DWI has emerged as a powerful tool for tumour response assessment in various malignancies [101,102,103]. A plethora of studies have investigated the role of DWI in NAT response in rectal cancer [104,105,106]. Increased rates of pathological complete response fostered the development of more conservative treatment approaches, such as ‘watch-and-wait,’ increasing the quality of life without impacting overall survival [107,108]. This has increased the need for precise imaging response assessment, with rectal MRI playing a pivotal role in detecting CR or near-complete response (nCR). Combined T2-weighted imaging (T2WI) and DWI interpretation has demonstrated better performance in identifying complete response in locally advanced rectal cancer than assessing solely morphological sequences [109]. Adding DWI significantly enhances the diagnostic performance of MRI for restaging after CRT, with a sensitivity of 94%, specificity of 77%, positive predictive value of 88%, negative predictive value of 87%, and overall accuracy of 88% in distinguishing between residual tumour versus complete response [110]. A multicentre validation study involving twenty-two radiologists further demonstrated the value of DWI in post-treatment assessment [111]. 

In the post-NAT setting, detection of residual tumour is impeded by post-treatment fibrotic changes on T2WI, with a mean sensitivity of 50.4% in detecting complete response [112]. Fibrotic tissue also exhibits low signal on DWI in areas with high collagen content. Residual active tumour, characterised by high cellularity and reduced extracellular fluid, impedes the diffusion of water molecules and produces a high signal on DWI. However, there are caveats to consider when interpreting DWI signals, such as susceptibility artefacts (intralumenal air, haemorrhagic changes), low ADC values with reduced DWI signal, T2 shine-through, rectal wall folding, or suboptimal plane angulation [113]. Due to the growing evidence of DWI’s utility in restaging rectal cancer, ESGAR guidelines recommend including DWI in routine imaging protocols for rectal cancer, particularly in restaging after NAT, as it aids in differentiating between complete response and partial response [114]. Combined T2-weighted imaging (T2WI) and diffusion-weighted imaging (DWI) methods appear to be optimal for detecting complete response [115]. This has significant clinical implications, as it may shift patient management towards a non-surgical approach. While some data suggest that the value of DWI in detecting tumour recurrence is limited, with no significant improvement in diagnostic performance compared to conventional MRI, DWI may still be useful in cases of inconclusive T2 findings [116]. Additionally, DWI may help detection of local recurrence after transanal endoscopic microsurgery [117].

Newer studies have explored quantitative DWI data in restaging MRI, with ADC being proposed as an imaging biomarker for tumour response assessment. Numerous studies have reported an increase in ADC value and relative increase in ADC value (ΔADC) after NAT, likely due to cell membrane disruption and increased necrosis caused by radiation [4]. Despite extensive evidence demonstrating the significance of ADC as a response biomarker, threshold ADC values are not reproducible, and there is notable overlap between studies. Further prospective multicentre studies are needed for validation and standardisation of these findings. The value of measuring tumour volume on high b-value sequences has also been explored, with some studies suggesting that tumour volume delineation on DWI is more accurate in detecting complete response than delineation performed on T2 sequences [118].

Recent studies emphasise the potential of pre-NAT ADC values in predicting pathological response and downstaging after TNT [119]. Data suggest that tumours with lower ADC values correlate with necrotic changes, indicating a response to NAT [120,121,122,123]. It is suggested that tumours with high cellularity have better response, probably due to increased mitotic rate and tissue vascularity compared to tumours with low cellularity, such as mucinous histology, and necrotic tumours with reduced perfusion, which are more resistant to chemoradiation. Limited significant results are reported with regards to ADC values in correlation with histopathological features such as differentiation grade and the expression of immunochemical markers having an impact on overall survival and disease-free survival. The potential use of DWI in predicting tumour aggressiveness was explored more than 10 years ago [124]; however, more recent data show that ADC values do not correlate with pathological features [120,125]. Multiparametric studies combine DWI data with chemical shift-encoded sequences, histogram analysis, or intravoxel incoherent motion parameters in an attempt to predict pathologic aggressive features such as Ki-67 expression, with heterogenous results [126,127,128,129,130]. Therefore, until standardisation and validation in prospective studies of quantitative ADC data is acquired, the DWI use should be limited to qualitative assessment.

### 3.2. Dynamic Contrast Enhancement (DCE-MRI)

Dynamic contrast-enhanced (DCE) MRI offers dynamic insights into tumour vascular perfusion and permeability by evaluating signal intensity changes over time as the contrast medium washes into and out of the tumour’s extracellular space. Since tumour perfusion and oxygenation are dynamic processes, functional MRI techniques such as DCE may characterise in vivo temporal and spatial tumour heterogeneity more than ex vivo histopathological analysis [131]. In addition, neoformation vessels contain friable, highly permeable walls which increase contrast medium uptake readily detected on DCE. This may also play a role in tumour cell dissemination and development of distant metastases.

DCE-MRI can be assessed either quantitatively, by measuring perfusion on a voxel-by-voxel basis, or semi-quantitatively, by plotting a time–intensity curve to determine the time to peak enhancement or the area under the curve. K-trans is the most studied parameter, offering valuable insights into tumour biology, including aggressiveness and the degree of vascularisation in rectal cancer [132].

DCE-MRI is not routinely included in rectal cancer evaluation, and ESGAR guidelines do not include DCE-MRI in the standard protocol for rectal cancer MRI assessment. Whilst it has been proven to have a significant input in research, translation into the everyday clinical setting is still debated. However, there is consensus regarding the role of DCE-MRI in detection of residual tumour after neoadjuvant treatment and in mucinous histology assessment [2]. A recent study found that T2WI combined with DCE had the highest accuracy (80.60%) for evaluating mrT staging of rectal cancer after neoadjuvant therapy when compared to T2WI alone and T2WI combined with DWI [14].

Research indicates that DCE-MRI is useful in predicting and evaluating responses after NAT, showing that a high K-trans value before CRT and a significant decrease in K-trans after CRT can predict complete response [3]. Additional data suggest that the kinetic parameter ‘late slope’ obtained from DCE may predict response to neoadjuvant treatment [133]. Delta K-trans may be a potential imaging biomarker for pathological response [134]. However, prospective data on large cohorts, with consistent and reproducible results, are needed to consider including DCE in routine response assessment.

Perfusion DCE parameters also appear to predict aggressive histological features and may indicate response to treatment [135]. A significant correlation between K-trans and pathological tumour differentiation grades has also been documented [135]. One study shows that DCE parameters have predictive value, with significantly reduced distant disease-free survival associated with high K-trans values compared to DWI parameters. The higher risk of systemic failure correlated with K-trans may be a potential indication for additional systemic therapy. Histogram analysis of K-trans can also assess tumour heterogeneity, which is not considered in DWI evaluation [136].

Recent studies show that DCE improves the detection of EMVI, one of the most important prognostic factors in rectal cancer, with a sensitivity of 52.4% and specificity of 83% [137]. The correlation between histogram analysis and quantitative evaluation may be useful in detecting EMVI in equivocal mrEMVI cases [138,139]. Additionally, combining DCE with texture analysis has been suggested to improve the accuracy of EMVI detection [140].

## 4. Radiomics and Artificial Intelligence (AI)

Radiomics analyses quantitative (numerical) data embedded in DICOM files using computational algorithms to identify imaging biomarkers that are not visible to the radiologist’s eye. Radiomic features are based on artificial intelligence, using either coded models engineered by experts in the field or machine learning algorithms generated automatically, with minimal human intervention [5]. Machine learning (ML), another field of AI, can develop mathematical models using convolutional neural networks (CNNs) and deep learning (DL). Radiomics and ML can be combined to increase the precision of the results [141]. However, a major limitation in reproducing and comparing results is the lack of standardisation in defining radiomic features and image processing. Open platforms have been developed to address this issue [142]. Nevertheless, radiomic systems show promising results in multiple areas of rectal cancer, including staging, prediction of NAT response, prognosis, histopathological characteristics, and genetic footprint. They can measure a wide range of phenotypic characteristics, including shape and texture, which may reflect biological properties such as tumour heterogeneity [143]. Other AI-based systems may also contribute to automatic tumour segmentation and 3D reconstruction of lesions of interest [5]. 

During staging, various radiomic models focused on differentiation between T1/T2 from T3/T4 stages, namely tumours limited at the muscularis mucosa from the ones extending into the mesorectal fascia, derived from T2WI and DWI, including texture analysis [144,145,146]. Machine learning techniques have also been employed to enhance accuracy [141]. Evaluation of N staging has increasingly gained interest, as conventional imaging only applies size and morphologic criteria to assess lymph node involvement. Some studies have achieved good results using radiomic features derived from T2 and DWI [144,145] or collective features before and after NAT [147]. The use of faster CNNs has improved malignant lymph node detection, yielding better results compared to the radiologists’ [148]. Identification of histopathological characteristics has also been explored during cancer staging, with good results in predicting the degree of tumour differentiation [144,149,150]. Various models have been proposed to aid in EMVI detection using T2WI, DWI, and DCE features; however, conventional MRI sequences remain the most reliable for assessing EMVI status in clinical practice [151]. 

Prediction of response to NAT is of great importance, as it may influence patient management and foster the development of more personalised treatments. Current imaging biomarkers are limited in assessing the likelihood of tumour response; therefore it is hypothesised that analysing multiple radiomic characteristics would reach statistical significance. One study focused on predicting pathological complete response (pCR) using radiomic features based on T2WI, DWI, and a combination of the two in patients treated with NAT, using post-surgical histopathologic analyses as a reference [152]. Pang et al. combined deep learning techniques with models derived from T2WI to predict CR and validated the model on an external dataset [153]. Another model, based on mesorectal fat features, proved useful in predicting pCR [154].

A more complex approach was proposed in a study that combined MRI clinical features—such as tumour location, whole tumour volume, cranio-caudal extension, distance from the internal anal sphincter, mesorectal fascia infiltration, extramural vascular invasion, extramural depth of invasion, T-stage, and N-stage—with 1688 radiomic features based on T2WI in a machine learning algorithm to predict treatment response [155]. The study suggested that adding machine learning techniques allows for the analysis of data from ‘multiple points of view,’ using multidimensional approaches rather than the standard univariate approach. A radiopathomic model was proposed by Feng et al., which combined radiomic MRI features with pathomic nucleus features and pathomic microenvironment characteristics to predict pCR [156]. Based on these findings, it seems that complex MRI ‘clinical–radiomic’ machine learning models could be more effective in predicting treatment response to NAT in rectal cancer than radiomic features alone.

Radiogenomics is an emerging field that aims to identify the relevant genotypic features of tumours such as KRAS/NRAS/BRAF that guide targeted therapy. Recent studies correlated T2WI radiomic features with KRAS status, obtaining significant results in differentiating wild-type from mutated KRAS tumours [150,157,158]. Microsatellite instability (MSI) status also determines responses to adjuvant 5-fluorouracil and immunotherapy, some radiomic models aiming to predict MSI high status of tumours [159,160], with validation in external cohorts [161]. 

Prognostic factors such as extranodal extension, local recurrence, and distant metastases are crucial in clinical decision-making. Radiomics enhanced by deep learning has shown promising results in predicting distant metastases in locally advanced rectal cancer after neoadjuvant therapy (NAT), with the potential to identify high-risk patients [162,163]. Other approaches include the analysis of intratumoural and peritumoural features [67]. Radiomics may predict extranodal tumour extension in resectable cancer patients, which is more difficult to assess with conventional MRI, while also providing data on recurrence-free survival [79]. The detection of local tumour recurrence is another valuable endpoint in radiomics, which may distinguish tumour recurrence from non-recurrence lesions at the site of anastomosis [164].

A recent systematic review demonstrated significant input of delta-radiomics in several clinical aspects, such as differentiating benign from malignant tumours, assessing prognosis, and predicting treatment response by evaluating radiomic features over time [165].

Texture analysis, a component of radiomics, offers an objective, quantitative evaluation of tumour heterogeneity by analysing and interconnecting the pixel levels or grayscale voxels within an image. This may be considered a ‘virtual biopsy,’ aiming to build a standardised model using selected texture features to determine clinically relevant conclusions [166]. In rectal cancer imaging, texture analysis has been used in a variety of settings, such as staging [145,146], response assessment [167,168], and KRAS status [169]. Even though several studies propose texture analysis as a potential imaging biomarker, several limitations remain. These include image acquisition and reconstruction, region of interest segmentation, and the extraction of image features to reproduce and validate the results [170].

Radiomics has the potential to guide the watch-and-wait (WW) strategy by enhancing the evaluation of restaging rectal MRI, specifically to identify complete responders, which is crucial for determining patient eligibility for this approach. An MRI-based radiomic model reached sensitivity of 71% and specificity of 88% in predicting good responders [171]. Another radiomics signature improved the accuracy of the mrTRG score in predicting response to NAT, achieving a sensitivity of 85%, specificity of 67%, PPV of 36%, and NPV of 95% for predicting pCR, and a sensitivity of 88%, specificity of 85%, PPV of 35%, and NPV of 96% for predicting ymrTRG 1-2 [172]. Detection of persistent EMVI and CRM involvement is often hindered by post-treatment changes; therefore, radiomics may enhance the identification of these features [151,173]. In addition, improving the accuracy of tumour deposits and malignant lymph nodes detection would be of values, as conventional MRI is limited in assessing these features [174,175]. Table 1 presents the wide range of radiomics applications, summarizing recent studies on MRI radiomics, including their assessed features and primary endpoints.

The ongoing collaboration of ACO/ARO/AIO-18.1, Janus Rectal Cancer, and ENSEMBLE trials aims to integrate multi-omics data for developing precision treatment models for LARC. By leveraging artificial intelligence, multi-omics integration enhances understanding of tumour heterogeneity, supporting accurate diagnosis and personalized therapy. Advanced supercomputing systems enable the combination of the world’s largest clinical image dataset with clinical and omics information, providing a robust computational environment for data analysis [178]. 

Whilst MRI-based radiomics provides valuable data mined from medical images, significant limitations still impede its clinical translation, including technical requirements, standardization, model reproducibility, and a lack of clinical validation [179]. Future perspectives may involve combining clinical data, validated imaging biomarkers, and radiomics, which appears to be a more feasible approach for clinical practice.

## 5. The Emerging Role of MRI in ‘Watch-And-Wait’ Strategies

Total neoadjuvant treatment (TNT) in locally advanced rectal cancer has triggered a paradigm shift from standard TME surgery. The advent of TNT has significantly improved the rates of pathological complete response in patients with locally advanced rectal cancer, as demonstrated by robust prospective studies (CAO/ARO/AIO-12, STELLAR, PRODIGE-23, and RAPIDO). This has paved the way for less invasive approaches, such as local excision and close follow-up without surgical intervention, reducing the morbidity associated with surgery without impacting overall survival [107].

The recently published long-term results of the prospective clinical trial known as ‘Organ Preservation in patients with Rectal Adenocarcinoma (OPRA)’ have the potential to revolutionise traditional paradigm of TME surgery. The study aimed to evaluate prognosis of ‘watch-and-wait’ (WW) approach in patients obtaining CR or nCR after TNT for locally advanced rectal cancer [180]. The trial was conducted across 18 specialised centres, where approximately half of the patients benefited from the organ preservation approach. It concluded that deferred surgery and active surveillance are possible for selected patients without compromising disease-free survival. 

An important challenge remains in defining CR to ensure a safe selection of patients amenable to WW. In line with previous research, a combination of digital rectal examination (DRE), endoscopy and rectal MRI (T2WI and DWI) was used, which appears to correctly identify 98% of patients with CR [181]. Clinical complete response was defined as the absence of residual tumour on all clinical, endoscopic, and imaging assessments. The endoluminal tumour component is readily assessed using DRE and endoscopy, whereas MRI is key for assessing the intramural component. Complete response on MRI includes the absence of intermediate tumour signal on T2WI, predominant fibrotic changes, and no signal on DWI sequences. nCR is defined by the persistence of tumour signal alongside fibrotic changes within the tumour bed and borderline or suspicious lymph nodes. Regarding lymph nodes, morphological criteria based on T2WI remain the standard. Organ preservation can be proposed only for negative lymph nodes. Previously, positive lymph nodes needed to completely disappear for the patient to be considered tumour-free. For persistent lymph nodes, imaging alone cannot reliably rule out residual tumour; therefore, TME surgery is a better option in these cases. The reassessment was conducted eight weeks, rather than six weeks, after the end of NAT, as evidence showed an increased rate of complete clinical response (cCR) with a longer reassessment interval after TNT completion [182]. Deferring reassessment by two weeks increases the percentage of patients suitable for a WW approach, as some may exhibit a delayed response to TNT.

Local recurrence is a major concern in organ-preservation approaches. Up to 34% of patients develop local relapse, with 97% occurring within the first two years of active surveillance [183]. Frequent reassessments are necessary to ensure early detection of tumour regrowth. The OPRA trial recommended DRE and endoscopy every four months, with MRI assessments every six months and CT scans of the chest, abdomen, and pelvis performed annually for the first two years, followed by longer intervals in the subsequent three years. This approach accounts for the higher likelihood of tumour recurrence in the first two years. Salvage TME surgery was indicated for patients who developed local tumour regrowth. The OPRA trial results indicated no significant difference in DFS between patients who underwent immediate TME and those who received salvage TME [183].

Residual lymph node disease remains a considerable limitation of MRI assessment. Although lymph node response often mirrors that of the primary tumour, there is still a significant risk of residual nodal disease in patients with a complete response, with one study reporting a rate as high as 17% [184].

The WW paradigm has the potential to benefit a significant proportion of rectal cancer patients with up to 76% reported in the OPRA trial, a figure considerably higher than the previously reported complete response rates of 27% [183]. This approach contributes to significantly reduced morbidity and improved quality of life. MRI has a limited sensitivity in detecting CR, especially in early reassessment, mainly due to mixed T2 signals, irregular fibrosis, and restricted diffusion [185]. One study reports up to 44% MRI overstaging after NAT, potentially denying conservative approach for these patients [186]. Therefore, reassessment should not rely solely on imaging but should be combined with clinical and endoscopic evaluation. Identification of gross residual disease that is amenable to surgical resection should be prioritized, distinguishing these cases from potential complete responders who may benefit from further clinical and endoscopic assessments to confirm CR.

Standardised pre- and post-treatment MRI reporting would facilitate patient selection for WW approach. Standardised tumour regression proformas, such as those used in clinical trials, contribute to the high number of patients eligible for organ preservation [183]. Including complete and relevant information in radiology reports is crucial to ensuring optimal therapeutic decisions in routine clinical practice. A multicentre study highlights that standardised reporting increased the completeness of oncologic imaging staging from 48.7% to 87.3% [8]. 

## 6. Future Directions

The paradigm shift from traditional TME surgery to organ-preservation strategies, driven by the advent of total neoadjuvant treatment (TNT), has made predictive and prognostic imaging biomarkers essential for optimizing the rectal cancer treatment strategy. Future research should address the issue of MRI overstaging after NAT, which has the potential to deny patients the WW approach. Standardisation of MRI reporting in post-NAT setting is crucial to ensure consistent and reliable patient selection for non-surgical management. The benefits of tumour regression proformas used in clinical trials, could be translated into MRI proformas for post-NAT response assessment designed for clinical practice.

Building on the promising insights from functional imaging and radiomics, particularly when integrated with deep learning, future research should prioritise the standardisation and reproducibility of models to fully explore the translational potential of radiomics in clinical practice. This would support wider acceptability of radiomics and increased adoption in routine clinical practice. 

## 7. Conclusions

Over the last decade, the management of rectal cancer has significantly broadened, with a crucial paradigm shift from conventional TME surgery to organ-preservation strategies due to considerable increase in clinically complete response rates following TNT. The evolution of non-invasive approaches in rectal cancer has increased the demand for non-invasive imaging biomarkers to support decision-making. Morphological features such as CRM, EMVI, tumour deposits, mucinous differentiation, and mrTRG have consolidated their position in the clinical setting, extending beyond their established prognostic roles to encompass patient stratification and treatment response prediction (key MRI features are listed in Table 2). Functional imaging techniques are becoming increasingly widespread, with DWI already serving as an indispensable tool for tumour response assessment. Non-standard biomarkers, including DCE, radiomic and texture analysis, and AI-driven features, hold tremendous potential for offering deeper insights into tumour physiology, histology, and genomic characteristics, such as tumour vascularisation, aggressiveness, degree of differentiation, KRAS and MSI status, all of which impact therapy selection. Despite their promising results, the lack of standardisation and reproducibility remains a major impediment to their adoption in clinical practice. Nevertheless, these emerging technologies warrant further investigation, as they hold the potential to advance personalised patient care.

## Figures and Tables

**Table 1:** Recent publications in radiomics and their main outcomes.

Reference	Year	Assessed Feature	Main Outcome
Hamabe et al. [173]	2024	Tumour segmentation	Optimal MRI imaging conditions can improve the accuracy of mrAI tumour delineation, enabling it to provide feedback to radiologists without overestimating tumour stage.
Wei et al. [176]	2024	T-staging MRF status	The multiparametric deep-learning model has the potential to aid clinicians by offering more accurate and reliable preoperative T staging diagnoses.
Liang et al. [151]	2024	EMVI detection	MRI-based radiomic models have strong diagnostic value in detecting EMVI in rectal cancer patients. Further prospective, high-quality studies with larger sample sizes are needed to validate these findings.
Sun et al. [174]	2024	Tumour deposits	Combining rad-score (T2WI + ADC) with clinical factors may serve as a tool to predict the presence of tumour deposits (TDs) in rectal cancer patients.
Abbaspour et al. [175]	2024	Positive lymph nodes	Artificial intelligence-based radiomics demonstrates promising results in preoperative lymph node staging for colorectal cancer, with significant predictive performance.
Miranda et al. [172]	2023	mrTRG	MRI-based rad-score has comparable diagnostic performance to ymrTRG
Li et al. [177]	2024	pCR	The review highlights that MRI-based radiomics holds great potential for predicting pathological complete responses to NAT in patients with locally advanced rectal cancer.

**Table 2:** A list of important MRI features used in the clinical practice.

MRI Feature	Main Point
T-staging	The maximum tumour extension beyond the muscularis propria should be staged as <1 mm (T3a), 1–5 mm (T3b), 5–15 mm (T3c) and >15 mm (T3d).
mrCRM	Positive circumferential margin is defined as a distance of 1 mm or less between the tumour and the mesorectal fascia; the same criteria is applied for irregular lymph nodes.
EMVI	Intermediate signal within the mesorectal vessels, with a loss of normal hypointense flow void; additional features include irregular contours and increased calibre of the mesorectal vessels; should be documented both pre- and post-treatment;
Lymph nodes	Malignant lymph nodes criteria: short axis diameter ≥9 mm, 5–8 mm and ≥2 suspicious features, <5 mm and 3 suspicious features, all mucinous lymph nodes; morphologically suspicious features: round shape, irregular border, internal heterogenous signal;
Tumour deposits (N1c)	Tumour deposits follow the course of a venous channel compared to lymph nodes which are usually isolated within the mesorectal fat
Mucinous tumours	Mucin component is readily identified as high signal intensity on T2WI
Tumour response assessment (mrTRG)	mrTRG1: complete response or no signs of residual tumour; mrTRG2: good response: >75% dense fibrosis, minimal, if any intermediate signal; mrTRG3: moderate response: >50% fibrosis and visible intermediate signal; mrTRG4: poor response: minimal fibrosis within intermediate signal; mrTRG5: no response: no fibrosis, unchanged original tumour;
